# Epidemiologic Features of Kawasaki Disease in Japan: Results of the 2007–2008 Nationwide Survey

**DOI:** 10.2188/jea.JE20090180

**Published:** 2010-07-05

**Authors:** Yosikazu Nakamura, Mayumi Yashiro, Ritei Uehara, Atsuko Sadakane, Izumi Chihara, Yasuko Aoyama, Kazuhiko Kotani, Hiroshi Yanagawa

**Affiliations:** Department of Public Health, Jichi Medical University, Shimotsuke, Tochigi, Japan

**Keywords:** mucocutaneous lymph node syndrome, incidence, cardiovascular diseases, immunoglobulin, intravenous, epidemiology

## Abstract

**Background:**

The most recent epidemiologic features of Kawasaki disease (KD) are unknown.

**Methods:**

The 20th nationwide survey of KD was conducted in 2009, and included patients treated for the disease in 2007 and 2008. Hospitals specializing in pediatrics, and hospitals with pediatric departments and 100 or more beds, were asked to report all patients with KD during the 2 survey years.

**Results:**

From a total of 1540 departments and hospitals, 23 337 patients (11 581 in 2007 and 11 756 in 2008) were reported: 13 523 boys and 9814 girls. The annual incidence rates were 215.3 and 218.6 per 100 000 children aged 0–4 years in 2007 and 2008, respectively. These were the highest annual KD incidence rates ever recorded in Japan. The monthly number of patients peaked during the winter months; smaller increases were noted in the summer months. The age-specific incidence rate showed a monomodal distribution with a peak at age 9–11 months. The prevalences of both cardiac lesions during the acute phase of the disease and cardiac sequelae were higher among infants and older age groups.

**Conclusions:**

The incidence rate and number of patients with KD in Japan continue to increase.

## INTRODUCTION

Kawasaki disease (KD) is a syndrome of unknown cause. It typically affects infants and toddlers, and causes systemic vasculitis.^1^^,^^2^ Cardiac lesions, eg, coronary artery aneurysms, are a salient characteristic of the disease.^2^^–^^6^ The most serious cardiac lesions are giant coronary aneurysms (those with a diameter ≥8 mm on 2-dimensional echocardiography), for which the prognosis is unfavorable. Prevention of these aneurysms is the primary target for pediatricians treating patients with KD.

Since 1970, nationwide epidemiologic surveys of KD have been conducted in Japan nearly every 2 years, and several features of the disease have been revealed.^7^^–^^11^ The most recent previous survey, the 19th, included patients treated in 2005 and 2006, and revealed that both the annual number of patients and the incidence rate had increased linearly. If the trend were to continue, the annual incidence rate in 2008 would be higher than 200 per 100 000 population younger than 5 years.^10^

Herein, we report the results of the latest nationwide survey, for KD patients treated in 2007 and 2008.

## METHODS

We conducted a retrospective survey of patients with KD visiting target hospitals for treatment of acute KD during the 2-year period from January 2007 through December 2008. The medical facilities that were requested to participate in the survey were hospitals specializing in pediatrics and hospitals with a pediatric department and 100 or more beds. These criteria have been used since the first nationwide survey in 1970.^12^ Questionnaires and diagnostic guidelines prepared by the Japan Kawasaki Disease Research Committee^13^ were sent by mail to administrators in charge of the pediatric department of their respective hospitals in January 2009. The prepared list of hospitals for the survey was based on the “Listing of Hospitals 2003–2004” compiled by the Committee on Studies of Health Policies, Ministry of Health, Labour and Welfare, Japan, and was revised using newly received information. A total of 2150 facilities met the conditions stated above.

The patient information requested on the questionnaire was: address (municipality), sex, date of birth, date and day of illness at first hospital visit, days of illness when discharged from the hospital, diagnosis (typical definite, atypical definite, and suspected), intravenous immunoglobulin (IVIG) therapy, additional therapy if conducted (additional IVIG therapy, steroids, infliximab, and immunosuppressive agents), recurrences, history of KD in patient’s siblings and parents, cardiac lesions, and complications other than cardiac lesions such as arthralgia or arthritis, aseptic meningitis, hepatic abnormalities (serum aspartate aminotransferase ≥50 IU/L and/or alanine aminotransferase ≥50 IU/L), gallbladder swelling, paralytic ileus, facial nerve palsy, and disseminated intravascular coagulation (DIC). Acute cardiac lesions were defined as those that developed within 1 month of onset (acute lesions); cardiac sequelae were defined as those that persisted beyond 1 month after onset. Almost all patients were diagnosed on the basis of 2-dimensional echocardiography.

After checking for possible inconsistencies on the questionnaires, the forms were sent back to the respondents to correct any errors. The incidence rates were based on the population data used in the vital statistics of Japan.^14^ The Ethical Board of Jichi Medical University approved this survey (November 11, 2008, No. 08-39).

## RESULTS

Of the 2150 invitations sent requesting participation in the survey, 48 were returned because the pediatric department or the institution itself had closed. Of the remaining 2102 departments, 1540 (73.3%) responded to the survey and reported a total of 23 337 patients (11 581 in 2007 and 11 756 in 2008). There were 13 523 male patients and 9814 female patients. The average annual incidence rate for the observed 2-year period was 216.9 per 100 000 children aged 0–4 years (245.4 for boys and 187.0 for girls).

**Figure 1.**
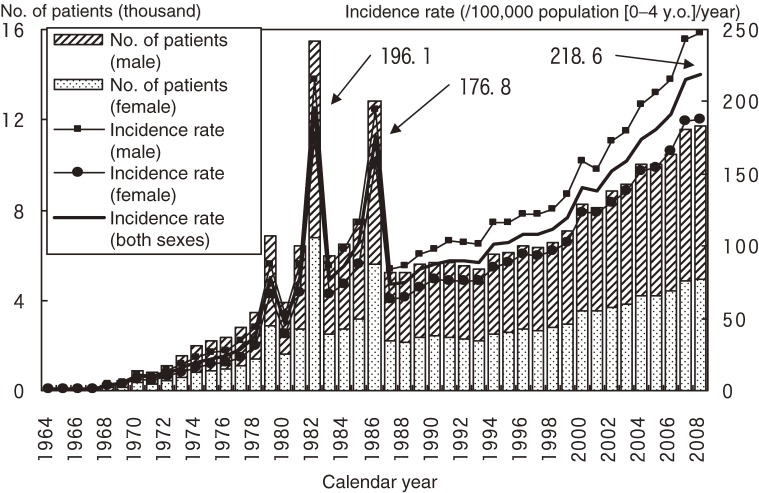
The number of patients with Kawasaki disease and incidence rate in Japan, by calendar year.

The annual numbers of patients with KD and the incidence rates in the 20 nationwide surveys, including this one, are shown in Figure 1. As previously reported, there were 3 large nationwide epidemics of the disease in Japan, in 1979, 1982, and 1986. Since then, there has been no nationwide epidemic, but the number of patients started to increase in the mid-1990s. Because of the decrease in the birth rate in Japan, the incidence rate increased more rapidly than did the number of patients, reaching 218.6 per 100 000 children aged 0–4 years in 2008. This was the first time that the incidence rate was higher than 200, and it surpassed the rates observed in 1979, 1982, and 1986, when the epidemics occurred.

**Figure 2.**
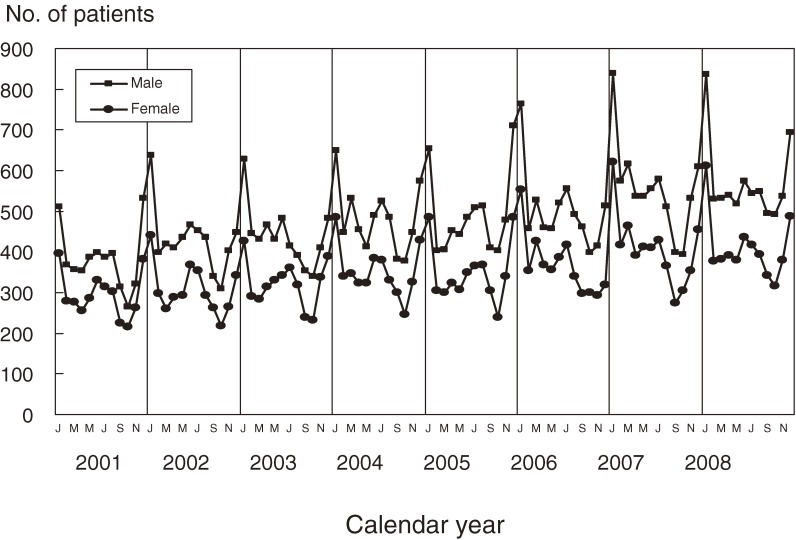
The number of patients with Kawasaki disease in Japan by month, 2001–2008.

Trends in the monthly number of patients observed in the previous 4 nationwide surveys (17th to 20th) are shown in Figure 2. The number was highest during the winter months in all years. There were also smaller increases during the summer months.

**Figure 3.**
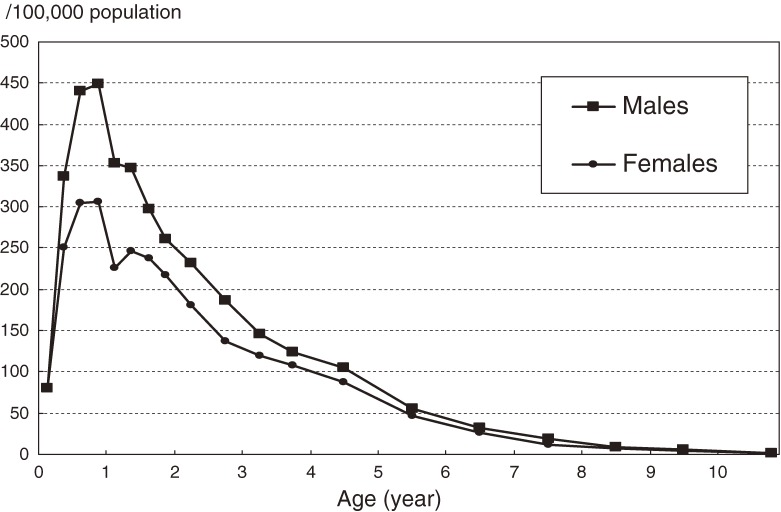
Age-specific annual incidence rate of Kawasaki disease in Japan, 2007–2008.

Age-specific incidence rates by sex are shown in Figure 3. As in previous surveys, the incidence rate was highest among children aged 6–11 months, after which it gradually decreased with advancing age.

Of the 23 337 patients reported, 18 620 (79.8%) were typical definite cases (patients with 5 or 6 of the symptoms specified in the diagnostic guidelines for KD), 648 (2.8%) were atypical definite cases (4 of the 6 symptoms plus coronary aneurysms including dilatation), and 4069 (17.4%) were suspected cases (those who did not satisfy the diagnostic criteria, but were suspected as having KD by the pediatricians reporting the cases). Of the 4069 suspected cases, 2661 (65.4%) had 4 of the 6 principal symptoms, 1063 (26.1%) had 3, 239 (5.9%) had 2, and 32 (0.8%) had 1.

The number of patients with a sibling affected by KD was 326 (1.4%); 165 (0.7%) patients had at least 1 parent with a history of KD. There were 823 (3.5%) recurrent cases. Of the 23 337 patients reported, 6 died.

**Figure 4.**
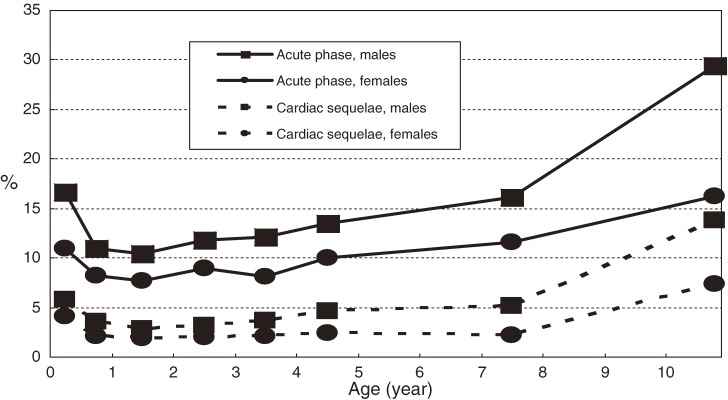
Age-specific prevalence of cardiac lesions and sequelae due to Kawasaki disease in Japan, 2007–2008.

During the acute phase, 2577 (11.0%) patients had (a) cardiac lesion(s): 58 (0.25%) had giant coronary aneurysms, 282 (1.21%) had coronary aneurysms less than 8 mm in diameter, 1992 (8.54%) had coronary dilatations, 8 (0.03%) had coronary stenoses, 3 (0.01%) had myocardial infarctions, and 383 (1.64%) had valvular lesions. A total of 746 patients (3.2%) had cardiac sequelae 1 month after the onset of KD: 59 (0.25%) had giant coronary aneurysms, 188 (0.81%) had coronary aneurysms less than 8 mm in diameter, 435 (1.86%) had coronary dilatations, 5 (0.02%) had coronary stenoses, 2 (0.01%) had myocardial infarctions, and 114 (0.49%) had valvular lesions. As shown in Figure 4, cardiac abnormalities were more prevalent in boys than in girls, and in infants and older children (as compared with children aged 1–4 years).

Of the patients reported, 20 313 (87.0%) received IVIG therapy. Of these, 3351 (16.5%) received additional IVIG therapy, 1173 (5.0%) were treated with steroids, 81 (0.35%) received infliximab, and 54 (0.23%) were treated with immunosuppressive agents.

Regarding patients with complications other than cardiac lesions, 1.13% had arthralgia or arthritis, 0.55% had aseptic meningitis, 27.4% had hepatic lesions, 1.62% had gallbladder swelling, 0.45% had paralytic ileus, 0.0% (1 patient) had facial nerve palsy, and 0.08% had DIC.

## DISCUSSION

We presented the results of the 20th Nationwide Survey of Kawasaki Disease in Japan, which highlighted the most recent epidemiologic features of the disease. Since 1970, the nationwide surveys have been conducted almost every 2 years.^7^^–^^11^ As shown in Figure 1, the number of patients and the incidence rate have dramatically increased since the mid-1990s. Even though there has been no nationwide epidemic of KD since 1986, the annual incidence rates in 2007 and 2008 were higher than those in the years of nationwide epidemics. Because the etiology of KD remains unknown, the reasons for these increases are also unclear. This increase is of concern and highlights the need for continued observation of the epidemiologic features of KD in Japan. Moreover, the results should motivate researchers to hasten their efforts to identify the cause of this disease.

The response rate of the survey was 73.3%, after 2 reminders were sent. Therefore, the actual number of patients was higher than that reported. However, data suggest that the real figures are at most 10% higher than the values we have reported.^15^ We asked the departments and hospitals to respond to the survey even if they had not treated a KD patient during the 2-year period of the survey. However, despite this request, many of the nonresponding hospitals might have elected not to participate in the survey because they had treated no KD patients. Therefore, the underestimation is approximately 10%, despite a participation rate of only 70%.

There are factors that can potentially distort the reporting of chronological trends in KD. One of these factors is response rate, which was 73.3% in the current survey. The response rate was 70.7% for the 19th survey (2005–2006),^10^ 70.1% for the 18th survey (2003–2004),^9^ 68.0% for the 17th survey (2001–2002),^8^ 66.5% for the 16th survey (1999–2000),^8^ 68.5% for the 15th survey (1997–1998),^16^ and 67.7% for the 14th survey (1995–1996).^16^ As is evident, the response rates were similar. Thus, we do not believe that changes in the response rate affected the analysis of chronological change. Another issue would be a change in diagnostic criteria. In the current survey, the Fifth Revised Version of the Diagnostic Guidelines of Kawasaki Disease (2002) was used. Although the guidelines have been revised to account for the increased understanding of KD since the first nationwide survey in 1970, the principal points have not changed.^13^ Therefore, the revision of the guidelines was unlikely to affect reporting of chronological changes. If the number of suspected cases increased, the clinical features of KD may also have changed. The proportion of suspected cases was 17.4% in the current survey, 14.3% in the 19th survey,^10^ and 13.6% in the 18th survey.^9^ Although there are no relevant data before the 18th survey, we do not believe that the proportion of suspected cases has increased to an extent that would affect the increases in the annual numbers of patients and incidence rates.

Even though the etiology of KD is unknown, the epidemiologic data suggest a relationship between the onset of the disease and infection.^17^ One trend suggesting an infectious trigger is seasonal variation in the disease. As shown in Figure 2, the number of patients is always higher in winter. In addition, smaller peaks were observed in summer. Perhaps infectious agents—one prevalent in winter and the other in summer—triggered the onset of KD. Seasonal variations differ among countries and areas, even in the same hemisphere, whether north or south.^18^ If the responsible infectious agents differ among countries and areas, this would explain variation in seasonal patterns among countries.

Regarding the age-specific incidence rate curve shown in Figure 3, a monomodal incidence rate curve was observed in the current survey, which indicates that there may be a relationship between disease occurrence and an infectious agent, in addition to the seasonal variation.^17^ The low incidence rate just after birth might be due to the presence of passive immunity conferred from mothers, and the decrease after 1 year of age might be caused by herd immunity.

Cardiac lesions are of great concern in KD. Fortunately, the proportion of patients with cardiac sequelae has decreased year by year. The proportion was 7.0% in the 15th nationwide survey in 1997–1998,^19^ 5.9% in the 16th (1999–2000),^8^ 5.0% in the 17th (2001–2002),^8^ 4.4% in the 18th (2003–2004),^9^ and 3.8% in the 19th survey.^10^ The proportion had been greater than 10% in the early 1990s.^20^ This improvement is due to progress in the diagnosis of KD, in identification of cardiac lesions, and in treatment, the core of which is IVIG therapy. Originally, the regimen for IVIG therapy was 200 mg or 400 mg per kilogram of body weight × 5 days, but this was changed to 2 g/kg for 1 day.^21^^,^^22^ The reduction in cardiac sequelae is partly due to this change. However, KD is the main cause of acquired heart disease in childhood both in Japan^23^ and in the United States,^24^ and treatment to prevent cardiac lesions must continue to progress. The higher proportion of lesions among infants might be due to their immature circulation system, which is affected by vasculitis caused by KD^25^; the higher proportion observed among older children might be due to the difficulty in diagnosing KD at this age, as some older children display an atypical clinical course.^26^ In this survey, 16.5% of patients treated with IVIG therapy received additional IVIG therapy, 5.0% were treated with steroids, and 0.4% and 0.2% were treated with infliximab and immunosuppressants, respectively. All of them may have been resistant to initial IVIG therapy during the acute phase. It is therefore important to identify the factors that predict such cases and the best treatment for such patients.

There are some limitations in the current survey. Because the etiology of the disease is unknown, there are no specific findings to aid in the diagnosis of the disease. Therefore, all the patients reported to the survey were diagnosed by pediatricians according to the diagnostic guidelines. Another problem is that some patients might have been reported by more than 1 hospital, as they may have been referred to another hospital due to the severity of the disease. In the 18th nationwide survey, 8.9% of the patients were referred from other hospitals, some of which were not included among the target hospitals for the survey, because of their small number of beds or the lack of pediatric departments; 4.9% of patients were referred to other facilities.^10^ The proportion of double registrations is likely to be lower than these figures, and the effects on the overall results are unlikely to be substantial.

In conclusion, the number of patients and incidence rate of KD in Japan continue to increase year by year, and cardiac lesions remain an important concern. The monitoring of KD should therefore be continued.

## References

[1] Burgner D , Harnden A Kawasaki disease: What is the epidemiology telling us about the etiology? Int J Infect Dis. 2005;9:185–94 10.1016/j.ijid.2005.03.002 15936970 PMC7110839

[2] Falcini F Kawasaki disease . Curr Opin Rheumatol. 2006;18:33–8 10.1097/01.bor.0000197998.50450.f6 16344617

[3] Pahlavan PS , Niroomand F Coronary artery aneurysm: a review . Clin Cardiol. 2006;29:439–43 10.1002/clc.4960291005 17063947 PMC6654377

[4] Imai Y , Sunagawa K , Ayusawa M , Miyashita M , Abe O , Suzuki J , A fatal case of ruptured giant coronary artery aneurysm . Eur J Pediatr. 2006;165:130–3 10.1007/s00431-005-0016-9 16215725

[5] Freeman AF , Crawford SE , Cornwall ML , Garcia FL , Shulman ST , Rowley AH Angiogenesis in fatal acute Kawasaki disease coronary artery and myocardium . Pediatr Cardiol. 2005;26:578–84 10.1007/s00246-005-0801-2 16132289

[6] Tsuda E , Hanatani A , Kurosaki K , Naito H , Echigo S Two young adults who had acute coronary syndrome after regression of coronary aneurysms caused by kawasaki disease in infancy . Pediatr Cardiol. 2006;27:372–5 10.1007/s00246-005-1233-8 16565902

[7] Yanagawa H, Nakamura Y, Yashiro M, Kawasaki T, eds. Epidemiology of Kawasaki disease: a 30-year achievement. Tokyo: Shindan-to-Chiryosha; 2004.

[8] Yanagawa H , Nakamura Y , Yashiro M , Uehara R , Oki I , Kayaba K Incidence of Kawasaki Disease in Japan: the Nationwide Surveys of 1999–2002 . Pediatr Int. 2006;48:356–61 10.1111/j.1442-200X.2006.02221.x 16911079

[9] Nakamura Y , Yashiro M , Uehara R , Oki I , Kayaba K , Yanagawa H Increasing incidence rate of Kawasaki disease in Japan: the nationwide survey . Pediatr Int. 2008;50:287–90 10.1111/j.1442-200X.2008.02572.x 18533938

[10] Nakamura Y , Yashiro M , Uehara R , Oki I , Watanabe M , Yanagawa H Epidemiologic features of Kawasaki disease in Japan: results from the nationwide survey in 2005–2006 . J Epidemiol. 2008;18:167–72 10.2188/jea.JE2008001 18635901 PMC4771586

[11] Nakamura Y , Yashiro M , Uehara R , Oki I , Watanabe M , Yanagawa H Monthly observation of the numbers of patients and incidence rates of Kawasaki disease in Japan: chronological and geographical observation from nationwide surveys . J Epidemiol. 2008;18:273–9 10.2188/jea.JE2008030 19075496 PMC4771612

[12] Yanagawa H. Summary of epidemiologic studies on Kawasaki disease in Japan. In: Yanagawa H, Nakamura Y, Yashiro M, Kawasaki T, eds. Epidemiology of Kawasaki disease: a 30-year achievement. Tokyo: Shindan-to-Chiryosha; 2004. p. 33–44.

[13] Yanagawa H, Sonobe T. Cahnges in the diagnostic guidelines for Kawasaki disease. In: Yanagawa H, Nakamura Y, Yashiro M, Kawasaki T, eds. Epidemiology of Kawasaki disease: a 30-year achievement. Tokyo: Shindan-to-Chiryosha; 2004. p. 24–32.

[14] Statistics and Information Department, Minister’s Secretariat, ministry of health, Labour and Welfare. Vital statistics of Japan 2007, volume 1. Tokyo: Health and Welafare Statistics Association; 2009. p. 481–3.

[15] Watanabe T , Oki I , Ojima T , Nakamura Y , Yanagawa H An analysis of the number of patients with Kawasaki disease in Tochigi: using the data of the nationwide epidemiologic incidence survey and the public aid in Tochigi prefecture . Nippon Shonika Gakkai Zasshi. 2002;106:1892–5 (in Japanese)

[16] Yashiro M, Yanagawa H. Database construction for information on patients with Kawasaki disease. In: Yanagawa H, Nakamura Y, Yashiro M, Kawasaki T, eds. Epidemiology of Kawasaki disease: a 30-year achievement. Tokyo: Shindan-to-Chiryosha; 2004. p. 57–77.

[17] Shigematsu I. Significance and future problems of epidemiologic studies on Kawasaki disease. In: Yanagawa H, Nakamura Y, Yashiro M, Kawasaki T, eds. Epidemiology of Kawasaki disease: a 30-year achievement. Tokyo: Shindan-to-Chiryosha; 2004. p. 1–5.

[18] Wu MH , Nakamura Y , Burns JC , Rowley AH , Takahashi K , Newburger JW , State-of-the-art basic and clinical science of Kawasaki disease: the 9th International Kawasaki Disease Symposium 10–12 April 2008, Taipei, Taiwan . Pediatr Health. 2008;2:405–9 10.2217/17455111.2.4.405

[19] Yanagawa H , Nakamura Y , Yashiro M , Oki I , Hirata S , Zhang T , Incidence survey of Kawasaki disease in 1997 and 1998 in Japan . Pediatrics. 2001;107:e33 10.1542/peds.107.3.e33 11230614

[20] Yanagawa H , Nakamura Y , Yashiro M , Ojima T , Koyanagi H , Kawasaki T Update of the epidemiology of Kawasaki disease in Japan: from the results of 1993–94 nationwide survey . J Epidemiol. 1996;6:148–57 8952219 10.2188/jea.6.148

[21] Academic Committee of the Japanese Society of pediatric Cardiology and Cardiac Surgery Guidelines for treatment of Kawasaki disease in acute phase . Nippon Shoni Junkanki Gakkai Zasshi (Pediatr Cardiol Cardiac Surg) 2004;20:54–62 (in Japanese).

[22] Freeman AF , Shulman ST Kawasaki disease: summary of the American Heart Association Guidelines . Am Fam Physician. 2006;74:1141–8 17039750

[23] Sonobe T A summary of the epidemiologic surveys on Kawasaki disease conducted over 30 years . Jpn Med Assoc J. 2005;48:30–3

[24] Shulman ST , Rowley AH Advances in Kawasaki disease . Eur J Pediatr. 2004;163:285–91 10.1007/s00431-004-1431-z 15346907

[25] Rosenfeld EA , Corydon KE , Shulman ST Kawasaki disease in infants less than one year of age . J Pediatr. 1995;126:524–9 10.1016/S0022-3476(95)70344-6 7699529

[26] Muta H , Ishii M , Sakaue T , Egami K , Furui J , Sugahara Y , Older age is a risk factor for the development of cardiovascular sequelae in Kawasaki disease . Pediatrics. 2004;114:751–4 10.1542/peds.2003-0118-F 15342849

